# Sensing User Intent: An LLM-Powered Agent for On-the-Fly Personalized Virtual Space Construction from UAV Sensor Data

**DOI:** 10.3390/s25247610

**Published:** 2025-12-15

**Authors:** Sanbi Luo

**Affiliations:** Software Engineering Center, Chinese Academy of Sciences, Beijing 100190, China; luosanbi@sec.ac.cn

**Keywords:** LLM agents, user intent sensing, dynamic content generation, hybrid IDC-RAG, UAV sensor data

## Abstract

The proliferation of Unmanned Aerial Vehicles (UAVs) enables the large-scale collection of ecological data, yet translating this dynamic sensor data into engaging, personalized public experiences remains a significant challenge. Existing solutions fall short: static exhibitions lack adaptability, while general-purpose LLM agents struggle with real-time responsiveness and reliability. To address this, we introduce CurationAgent, a novel intelligent agent built upon the State-Gated Agent Architecture (SGAA). Its core innovation is an advanced hybrid curation pipeline that synergizes Retrieval-Augmented Generation (RAG) for broad semantic recall with an Intent-Driven Curation (IDC) Funnel for precise intent formalization and narrative synthesis. This hybrid model robustly translates user intent into a curated, multi-modal narrative. We validate this framework in a proof-of-concept virtual exhibition of the Lalu Wetland’s biodiversity. Our comprehensive evaluation demonstrates that CurationAgent is significantly more responsive (1512 ms vs. 4301 ms), reliable (95% vs. 57% task success), and precise (85.5% vs. 52.7% query precision) than standard agent architectures. Furthermore, a user study with 27 participants confirmed our system leads to measurably higher user engagement. This work contributes a robust and responsive agent architecture that validates a new paradigm for interactive systems, shifting from passive information retrieval to active, partnered experience curation.

## 1. Introduction

Virtual exhibitions have been widely adopted as a powerful medium for science communication and cultural heritage presentation [1,2]. However, the dominant paradigm for most existing virtual exhibitions remains a “one-size-fits-all”, static approach [3]. In this model, the layout of the exhibition space, the content of the exhibits, and the narrative paths are all pre-authored and hard-coded by developers. While such works have achieved a high degree of visual fidelity, their fundamental limitation lies in their lack of adaptability to individual user differences. All users, regardless of their knowledge background, interests, or exploratory intent, are passively presented with the same fixed information architecture. This model fails to respond to the user’s immediate curiosity, thereby limiting the potential for deep engagement and personalized learning [4,5]. This challenge is profoundly amplified by the concurrent revolution in remote sensing technologies, particularly Unmanned Aerial Vehicles (UAVs), which generate vast and frequently updated biodiversity data [6]. Recent advancements in multi-UAV cooperation, such as distributed localization under security threats [7] and reliable data collection in IoT networks [8], further ensure the robustness and fidelity of such environmental sensing sources. A critical contradiction has thus emerged: a dynamic, ever-expanding ocean of real-world sensor data is being funneled into a static, immutable pond of information [9]. The static paradigm is thus failing on two fronts: it cannot meet the user’s need for personalization, and it cannot keep pace with the scale and dynamism of modern scientific data.

Prior to the advent of Large Language Models (LLMs), the academic community attempted to address these limitations through various computational approaches, yet a comprehensive solution remained elusive. In the pursuit of personalization, research in virtual museums explored user profiling and collaborative filtering to recommend exhibits [4,10]. While valuable, these methods offer a form of “shallow personalization,” adapting to a user’s historical behavior or declared interests rather than their immediate, in-the-moment conversational intent. On the content generation front, Procedural Content Generation (PCG) techniques have been employed to create diverse virtual environments [11]. However, traditional PCG typically relies on complex, hand-authored rule-based systems that, while powerful for generating structural variations, lack the semantic understanding required to interpret and respond to the nuanced, high-level goals expressed in a user’s natural language [12]. These prior approaches, therefore, only partially addressed the problem, highlighting the need for a disruptive technology capable of both deep semantic understanding and real-time content generation.

The emergence of LLMs offers a new and powerful paradigm [13]. Building intelligent agents upon LLMs has become a frontier of research, with frameworks like ReAct demonstrating impressive capabilities in complex reasoning and planning [14]. However, a fundamental mismatch exists between the design philosophy of these General-Purpose Reasoning Agents and the stringent requirements of Domain-Specific Real-Time Interaction. General-purpose frameworks, in their pursuit of broad applicability, often employ complex, multi-step “thought-action” loops that necessitate multiple sequential calls to the LLM [15,16]. While effective for solving open-ended problems, this process introduces significant, often multi-second, latency. Such delays are unacceptable in real-time Human-Computer Interaction (HCI) scenarios that demand immediate visual feedback, where a user’s fleeting curiosity must be captured and responded to in milliseconds, not seconds [17]. A novel agent architecture is therefore required—one that eschews complex, multi-step reasoning in favor of a highly responsive, state-aware, single-pass intent translation mechanism [18].

To address these challenges, we propose CurationAgent, a novel system built upon the State-Gated Agent Architecture (SGAA), a novel framework designed specifically to harness the capabilities of LLMs for real-time, personalized virtual space construction. The core of the SGAA is a ‘fast and slow path’ decision-making system, drawing theoretical inspiration from Dual Process Theory [19]. This theory posits that cognition consists of two distinct systems: System 1 (fast, automatic, instinctive) and System 2 (slow, deliberative, logical). By mapping high-frequency interactions to a deterministic FSM (mimicking System 1) and complex intent reasoning to an LLM (mimicking System 2), SGAA effectively operationalizes this cognitive efficiency within an artificial agent. While sharing the efficient dual-path philosophy of recent hierarchical agents [20], our architecture introduces a deterministic Finite State Machine (FSM) to enforce procedural guardrails, thereby directly resolving the reliability issues often faced by probabilistic routing. Its “fast path” employs a deterministic FSM and a Command Cache to handle high-frequency commands with millisecond-level latency and 100% reliability. The “slow path,” meanwhile, is intelligently activated under the FSM’s state-gated control, invoking our Intent-Driven Curation (IDC) Funnel. The IDC Funnel serves as the agent’s creative core. Distinct from recent personalization frameworks that primarily augment LLMs with retrieved interaction history [21], our approach uses the LLM to first formalize a user’s ambiguous natural language intent into a precise, machine-executable structured query into a precise, machine-executable structured query, and then creatively synthesize the retrieved data points into a multi-modal experience with generated narrative and spatial layout suggestions. Our comprehensive evaluation of this framework provides strong empirical evidence that it not only significantly outperforms traditional agents in performance and reliability, but more importantly, transforms the user experience by enabling a shift from passive information retrieval to active, personalized exploration, leading to higher user engagement.

The main contributions of this work are:

- SGAA Framework: A dual-path decision system for low-latency, reliable interaction, using FSM and Command Cache for high-frequency commands, and an LLM-driven IDC Funnel for complex queries;
- IDC Funnel: A multi-stage LLM-powered pipeline that converts user intent into structured queries and generates dynamic, personalized virtual experiences;
- Empirical Validation: The framework outperforms traditional agents in response time (1512 ms vs. 4301 ms), task success (95% vs. 57%), and query precision (85.5% vs. 52.7%). A user study confirmed improved user engagement.

## 2. Methodology: The Personalized Virtual Space Construction Framework

Our methodology is realized through a comprehensive framework that grounds a central intelligent agent in real-world sensor data and enables it to communicate its decisions to a rendering environment.

### 2.1. Overall Framework Overview

**Figure 1:**
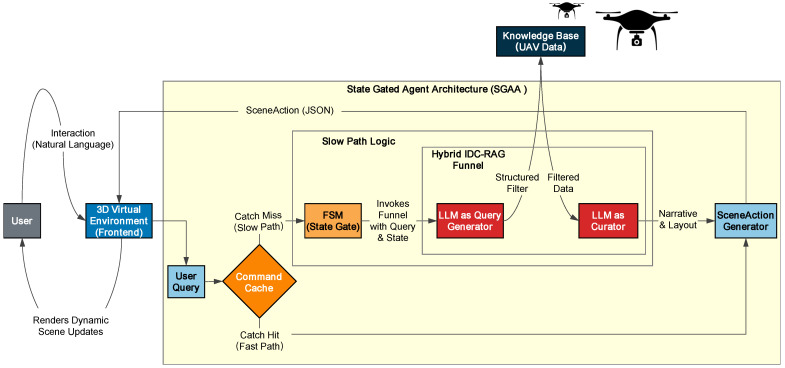
The Overall Architecture of CurationAgent. The system processes a user’s natural language query via the State-Gated Agent Architecture (SGAA). It utilizes a “fast path” (Command Cache) for high-frequency commands and a “slow path” featuring the Hybrid IDC-RAG Funnel to translate complex user intent into a dynamically generated, personalized virtual scene.

The framework follows a clear input-process-output pipeline (Figure 1). (1) Knowledge Base Construction: Raw data from UAV sensors is processed and structured into a multi-modal knowledge base. (2) The Agent’s Core Logic: Our primary contribution, where the agent’s memory and data funnel work in tandem to sense and process user intent. (3) The Decoupled Action Protocol: The agent’s decisions are translated into a standardized command format (SceneAction) that instructs the frontend on how to construct the virtual scene.

### 2.2. Grounding the Agent: Knowledge Base Preparation

The foundation of our system is a comprehensive, structured knowledge base derived from UAV sensor data. The construction of this knowledge base follows a systematic data processing pipeline, which transforms raw sensor data into a queryable, multi-modal library of assets. The primary steps of this pipeline, which involve processing photogrammetry data to create assets and enriching them with structured metadata, are formalized in Algorithm 1. This grounding stage is crucial, as it ensures that all of the agent’s subsequent reasoning and creative curation are based on a high-quality, verifiable, real-world dataset.

**Technical Implementation of Data Transformation and Indexing:** To specifically address the data transformation pipeline outlined in Algorithm 1, we implemented a streamlined process leveraging advanced open-source models:

- AI-Powered Structured Metadata Generation: For each identified asset (e.g., a bird species), we automated the enrichment of sparse raw data into comprehensive JSON entries. This was achieved by prompting the Qwen2.5-72B-Instruct LLM [22] with precise system instructions to synthesize attributes such as scientific names, detailed descriptions, habitat, and conservation status from raw textual identifiers.
- Vector Embedding with Pre-trained Models: To enable the semantic retrieval crucial for the RAG component, we consolidated the textual attributes of each entry into a coherent string and transformed them into high-dimensional vector embeddings. We utilized BAAI/bge-large-zh-v1.5 [23], a specialized pre-trained model optimized for semantic similarity, to ensure high-fidelity representation of the domain-specific biodiversity data.
- Efficient Similarity Search via FAISS: The generated embeddings were indexed using the Facebook AI Similarity Search (FAISS) library [24]. Specifically, we employed vector similarity search, which transforms the collection of embeddings into an optimized data structure. This allows for highly efficient nearest-neighbor queries, enabling the system to dynamically match user intent with relevant knowledge base entries with millisecond-level latency. Critically, this standardized pipeline—spanning data ingestion, embedding generation (BAAI/bge), and vector indexing (FAISS)—serves as the foundational retrieval mechanism for both our proposed IDC-RAG framework and the ’Standard RAG’ baseline used in our comparative experiments (see Section 3).

**Algorithm 1** Knowledge Base Construction Pipeline
  1:  **function** BuildKnowledgeBase(raw_uav_data)  2:                                       ▹ Input: Raw sensor data (e.g., images from photogrammetry).  3:        *KnowledgeBase* ← new empty list  4:        *asset_library* ← ProcessPhotogrammetry(*raw_uav_data*)  ▹ Creates assets for bird species.  5:        **for all** asset in asset_library **do**  6:                     ▹ Create a structured entry by extracting and enriching data for each asset.  7:              entry← Create structured object with schema:          {              “id”: GenerateID(asset),              “common_name”: ExtractName(asset),              “image_urls”: FindImagePaths(asset),              “audio_url”: FindAudioPath(asset),              “metadata”: FetchMetadata(asset.name)       ▹ Fetches habitat, status, etc.          }  8:              KnowledgeBase.add(entry)  9:        **end for**10:        **return** KnowledgeBase11:  **end function**


### 2.3. The Agent’s Core Intelligence: SGAA and IDC Funnel

#### 2.3.1. The State-Gated Agent Architecture: A Framework for Robust, Real-Time Interaction

**Figure 2:**
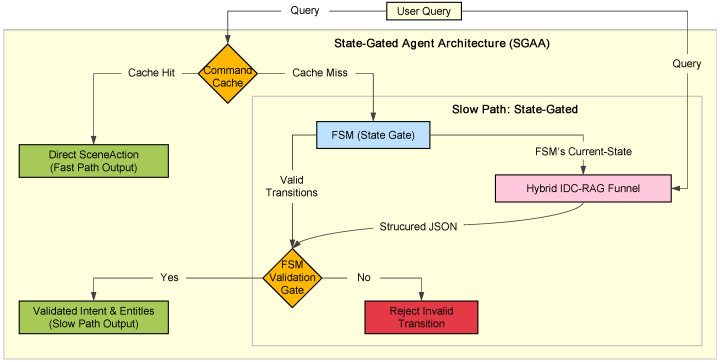
Overall SGAA Architecture.

At the heart of our agent lies the SGAA (Figure 2), a novel framework designed to orchestrate the agent’s decision-making process through a dual-system model. This architecture diverges from conventional, monolithic LLM-centric designs by segregating interaction handling into two distinct pathways: a deterministic “fast path” for high-frequency, predictable commands, and an adaptive “slow path” for complex, open-ended user intents. This architecture is realized through a lightweight hybrid memory model, where a deterministic FSM acts as the central “gate” controlling the flow of interaction, ensuring an optimal balance of responsiveness, reliability, and intelligent capability.

**1. The Deterministic “Fast Path”: FSM & Command Cache**

The “fast path” serves as the robust, instantaneous backbone of the interaction. It is comprised of two key components: an FSM and a Command Cache.

**FSM as the Interaction Skeleton:** We employ a simple, yet powerful, FSM to define and enforce the valid states and transitions of the task-oriented dialogue. For instance, in a virtual tour, states such as AWAITING_SELECTION, DISPLAYING_EXHIBIT, and IN_TOUR_MODE are explicitly defined. The transitions between these states are governed by a predefined set of SceneAction.action_type values (e.g., SHOW_INFO_SINGLE, SHOW_INFO_GROUP, GENERAL_RESPONSE), which serve as the FSM’s formal triggers. This deterministic structure acts as a “guardrail” for the interaction, making it logically impossible for the agent to enter an invalid state or perform an out-of-sequence action. In our implementation, this is realized through a state dictionary and conditional logic that validates every proposed state change, providing a formal guarantee of procedural integrity. For the “Fast Path,” these triggers are directly generated by matching cached user inputs to predefined SceneAction objects. For the “Slow Path,” they are derived from the LLM’s formalized intent by the backend logic after a multi-stage reasoning process. This contrasts sharply with monolithic, end-to-end approaches like the ReAct framework, where the agent’s entire reasoning and action trajectory is controlled by the LLM, making it susceptible to logical errors and conversational dead-ends.

**Command Cache for Instantaneous Responsiveness:** To handle simple, high-frequency user utterances (e.g., “hello,” “thank you”), we implement a Command Cache. This cache maintains a direct, hash-map-based mapping from normalized user inputs to predefined SceneAction responses. When a user’s input exactly matches an entry in the cache, the system bypasses the LLM entirely and returns the corresponding action within milliseconds. As demonstrated in our implementation, these cached responses carry a specific flag (is_fast_path) that instructs the user interface to handle them immediately without triggering a “thinking” state.

We deliberately chose a direct-mapping Command Cache over a more complex Semantic Cache (e.g., vector-based) for the fast path. This choice is fundamental to our architecture’s core principle of separating deterministic and probabilistic processing. The fast path is designed for commands where 100% accuracy and near-zero latency are paramount. While a semantic cache offers greater flexibility in understanding utterance variations, it reintroduces a layer of probabilistic uncertainty and computational overhead that is undesirable for this pathway. Instead, our SGAA architecture delegates all semantic understanding tasks to the LLM on the slow path, which is best equipped for that purpose. This clear division of labor ensures that our system’s intelligence is not compromised, but rather, is applied more efficiently and reliably.

**2. The Adaptive “Slow Path”: State-Gated LLM Understanding**

When a user’s input is not found in the Command Cache, the system escalates the request to the adaptive “slow path,” which leverages the sophisticated natural language understanding capabilities of an LLM. However, our SGAA framework utilizes the FSM’s current state as a powerful, high-signal contextual gate before invoking the model.

**State-Aware Prompt Engineering:** The prompt sent to the LLM is dynamically constructed and always includes the FSM’s current_state. For example, if the user says “show me something blue” while the FSM is in the AWAITING_SELECTION state, the prompt will frame the query within this specific context. This state-awareness acts as a powerful disambiguation mechanism. The same user utterance, if made in a DISPLAYING_EXHIBIT state, might be interpreted as a request to find blue elements within the current exhibit, rather than a new search query. By informing the LLM of the immediate task context, we significantly reduce ambiguity and constrain the LLM’s search space, leading to more accurate and relevant intent classification.

**Structured and Constrained LLM Output:** We do not permit the LLM to generate freeform text responses for intent recognition. Instead, we instruct it to return a structured JSON object that conforms to a predefined schema. This schema explicitly includes an intents field (a list of user goals like [“SHOW_INFO”], [“COUNT”]) and a filters object (containing search criteria). These fields represent the LLM’s semantic interpretation and formalization of the user’s natural language query. An example of the LLM’s structured JSON output is shown in Listing 1:

Crucially, the FSM trigger is determined by the backend logic’s interpretation of this LLM-formalized intent. The backend processes the LLM’s JSON output (e.g., “intents”: [“type”: “SHOW_INFO”], “filters”: “habitat”: “forest”) and, based on both the inferred intent and the success/failure of subsequent data retrieval, maps it to a concrete SceneAction.action_type value. These action_types (e.g., SHOW_INFO_GROUP, GENERAL_RESPONSE) are the actual triggers that the FSM uses to manage state transitions and frontend actions.

**Listing 1.** Example JSON Schema for LLM Intent Formalization. *Note:* This schema details the primary fields (intents, filters, count, layout) that the LLM is instructed to output. The action_type (FSM trigger) is derived by the backend from this structured intent and subsequent data retrieval results, not directly from the LLM output.

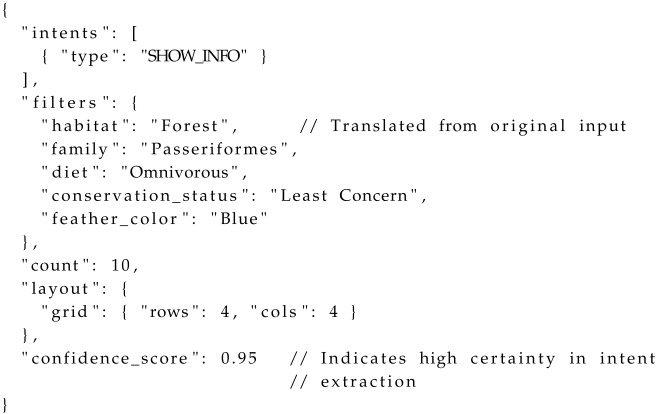



The FSM retains ultimate control and embodies a robust rejection logic:

- **Invalid or Unfulfillable Intents:** If the LLM’s output cannot be successfully parsed, or if the subsequent data retrieval (combining IDC and RAG paths) fails to identify any relevant exhibits based on the LLM’s formalized intent, the backend system does not generate an invalid FSM trigger. Instead, it explicitly constructs a SceneAction with action_type: “GENERAL_RESPONSE” and a user-facing explanatory message (e.g., “Sorry, no bird information found matching your description.”). This provides immediate feedback to the user regarding the unfulfilled request.
- **System Errors:** In cases of unexpected backend processing errors (e.g., LLM API failure, data processing exceptions), a SceneAction with action_type: “ERROR” and a generic apology is sent to the user. All such incidents are also thoroughly logged to the backend console for diagnostic purposes.
- **FSM State Maintenance:** Upon such a “rejection” (i.e., when a valid, actionable SceneAction.action_type corresponding to the user’s positive intent cannot be generated), the FSM maintains its current valid state. It does not attempt to transition to an undefined or error state. The frontend receives the GENERAL_RESPONSE or ERROR action, displays the feedback, and awaits further user input, ensuring the system’s stability, predictability, and a graceful user experience.

This transforms the LLM from an unpredictable “black box” controller into a powerful, yet constrained, semantic analysis engine. Ultimately, the FSM acts as the final gatekeeper, ensuring that only valid, state-consistent actions are executed, thus preserving the integrity of the interaction. The core logic of the agent’s slow path, which orchestrates intent recognition and content curation based on the FSM’s current state, is detailed in Algorithm 2.
**Algorithm 2** State-Gated Intent Processing and Curation  1:  **function** ProcessSlowPath(query,state,history,bird_data)  2:                                    ▹ This function is invoked on a “cache miss” from the fast path.  3:        **if** state is AWAITING_SELECTION **then**  4:              *prompt* ← BuildInitialIntentPrompt(*query*)  5:              structured_intent←LLM(prompt)           ▹ Uses the Initial Intent Recognition template  6:              precise_ids=FILTERDATABASE(bird_data,filters)(IDCPath)  7:              semantic_ids=SEARCH_VECTOR_DB(query)(RAGPath)  8:              final_ids=CONSOLIDATE_IDS(precise_ids,semantic_ids,filters)  9:        **else if** state is DISPLAYING_EXHIBIT **then**10:              prompt← BuildFollowUpIntentPrompt(*query*)11:              structured_intent←LLM(prompt)▹ Uses the Follow-up Intent Recognition template12:        **end if**13:        action←null14:        **if** structured_intent.intents contains “SHOW_INFO” **then**15:              *filtered_data* ← FILTERDATABASE(*bird_data*, *filters*)16:              *narrative_prompt* ← BUILDNARRATIVEPROMPT(*query*, *history*, *final_ids*)17:              curated_response←LLM(narrative_prompt)    ▹ Uses the Narrative Curation template18:              *action* ← CREATESCENEACTION(*curated_response*, *structured_intent.layout*)19:        **else**20:                                                          ▹ Handle other intents like COUNT, $LIST_{N}AMES$, etc.21:              *action* ← CREATESCENEACTION(null, null)22:        **end if**23:        **return** action24:  **end function**
25:  **function** BuildInitialIntentPrompt(query)26:        template←“Youareanexpertqueryanalysisagent...”                                        ▹ As per intent_prompt_template.txt27:        prompt←template.replace(“query”,query)28:        **return** prompt29:  **end function** 30:  **function** BuildNarrativePrompt(query,history,data)31:        template←“YouareanAIassistantactingasascenenarrator...”                ▹ As per prompt_template.txt32:        prompt←template.replace(...)                                      ▹ Injects query, history, and data33:        **return** prompt34:  **end function**

#### 2.3.2. The Hybrid IDC-RAG Funnel: A Multi-Stage Process for Robust Curation

**Figure 3:**
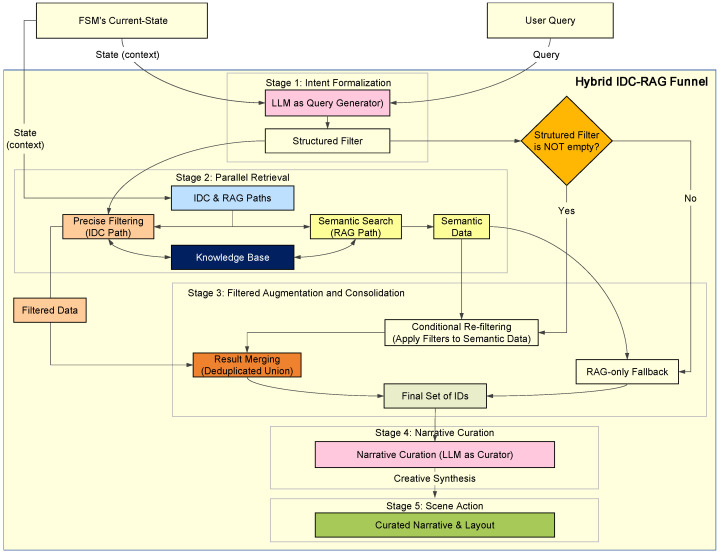
Overall IDC-RAG Architecture.

To translate a user’s high-level, often ambiguous, natural language intent into a structured, personalized virtual experience, we developed a novel multi-stage pipeline named the IDC (Figure 3) Funnel. This framework fundamentally differs from conventional Retrieval-Augmented Generation (RAG [25]) paradigms in its core objective, mechanism, and capabilities.

**The fundamental limitation of standard RAG lies in its mechanism:** its reliance on vector-based semantic search means it is probabilistic, not logical. RAG cannot make a definitive, binary decision (a “0 or 1” judgment) on whether an item perfectly satisfies a user’s logical constraints (e.g., “NOT endangered”). It can only return a ranking of results based on semantic similarity. This inability to distinguish semantic relevance from strict logical adherence is the primary reason it fails on complex queries, as demonstrated in our experiments (Section 3.2.3).

While RAG is designed to find and summarize similar answers, our IDC Funnel is engineered to decisively understand creative intent and precisely construct a curated experience. This process progressively formalizes the user’s intent, first into a machine-executable query, and second, into a human-perceptible narrative.

The Hybrid IDC-RAG Funnel is implemented (as depicted in Figure 3) through a granular multi-stage process to robustly integrate both precise filtering and semantic augmentation:

**Stage 1: Intent Formalization (LLM as Query Generator)**

The user’s natural language query is first processed by an LLM, acting as a “Query Generator”. Utilizing a state-aware prompt, the LLM’s primary task is to formalize the user’s ambiguous intent into a precise, machine-executable JSON object containing structured filters (e.g., “conservation_status”: “Endangered”, “family”: “Anatidae”). This stage is the core of the IDC component, translating high-level intent into actionable constraints. To enhance the robustness of intent formalization and provide a mechanism for distinguishing between broad queries and LLM failures, the LLM output schema explicitly includes a confidence_score (ranging from 0 to 1), indicating the model’s certainty in its extracted intent and filters. To enhance the robustness of intent formalization and provide a mechanism for distinguishing between broad queries and LLM failures, the LLM output schema explicitly includes a confidence_score (as detailed in Listing 1 in Section 2.3.1), indicating the model’s certainty in its extracted intent and filters.

**Stage 2: Parallel Retrieval**

Following intent formalization, two parallel retrieval mechanisms are activated:

- **Precise Filtering (IDC Path):** If structured filters were successfully extracted in Stage 1, these filters are applied directly to the knowledge base to identify a set of precisely matching bird IDs. This leverages IDC’s logical precision.
- **Semantic Search (RAG Path):** Concurrently, a vector-based semantic search is performed on the original user query. This retrieves a set of semantically related bird IDs based on their semantic similarity to the query, providing a broader set of potentially relevant entities.

**Stage 3: Filtered Augmentation and Consolidation**

This stage intelligently merges the results from the parallel retrieval paths:

- **Conditional Re-filtering:** Ensuring Logical Adherence with Semantic Relevance. If structured filters were present (i.e., extracted_filters is not empty), the semantically related bird IDs obtained from the RAG path are subjected to a secondary re-filtering process using the exact same precise IDC criteria. This step is critical for overcoming RAG’s probabilistic limitations and ensuring logical accuracy.
  To illustrate its value, consider the query: “Show me endangered blue birds.”
  1. In Stage 1, the LLM formalizes this into filters: “conservation_status”: “Endangered”, “feather_color”: “Blue”.
  2. In Stage 2, the RAG path might retrieve a broad set of semantically related birds. Due to vector proximity, this set could inadvertently include a “Blue-feathered” bird whose conservation_status is actually “Least Concern” (e.g., Blue Eared Pheasant), or an “Endangered” bird whose feather_color is “Green”. These are typical RAG “false positives”—semantically relevant but logically incorrect.
  3. The Conditional Re-filtering stage strictly applies the logic conservation_status=“Endangered”ANDfeather_color=“Blue” to all RAG-retrieved IDs. Any bird failing either condition is precisely eliminated.
  This process ensures that any semantically relevant entities from RAG also strictly adhere to the user’s explicit logical constraints, yielding a set of re-filtered semantic bird IDs. This mechanism dramatically improves the precision of the final results by eliminating logically incongruent entities that a pure RAG system might otherwise present.
- **Result Merging:** The final set of bird IDs for the narrative are then formed by taking the deduplicated union of the precisely matching bird IDs (from the initial IDC filtering) and the re-filtered semantic bird IDs (from the re-filtered RAG path). This strategy ensures that all deterministically matching entities are included, augmented by semantically relevant entities that also satisfy the precise filters.
- **RAG-only Fallback:** Differentiating Broad Queries from LLM Failures. If no structured filters were extracted in Stage 1 (extracted_filters is empty), the system differentiates between two scenarios based on the confidence_score from the LLM’s intent formalization:
  1. Broad Query (High Confidence): If extracted_filters is empty and the confidence_score is high (e.g., >0.7), it indicates that the user’s query was intentionally broad or general (e.g., “Show me some birds”), and no specific filters were needed. In this case, the system relies solely on the semantically related bird IDs from the RAG path as the final set of bird IDs for the narrative.
  2. LLM Failure (Low Confidence): If extracted_filters is empty and the confidence_score is low (e.g., <0.7), or if the LLM’s output was malformed, it signals that the LLM failed to understand or formalize the user’s intent. In this scenario, the system does not proceed with RAG fallback. Instead, it returns a SceneAction with action_type: “GENERAL_RESPONSE” containing an explicit error message (e.g., “Sorry, I could not understand your request. Please try rephrasing.”). Detailed error logs are generated, and the FSM maintains its current state, ensuring system stability.

**Stage 4: Narrative Curation (LLM as Curator)**

With the final set of relevant bird entities identified, an LLM, acting as a “Curator”, synthesizes this data. Using a dedicated narrative prompt, the LLM generates a coherent, engaging, and personalized multi-modal narrative that weaves together the discrete data points into a compelling story, rather than merely listing facts.

**Stage 5: Scene Action Generation**

Finally, the curated narrative and the detailed information for each selected bird are packaged into a standardized SceneAction object. This object, which includes layout suggestions and asset URLs, serves as a decoupled protocol to instruct the frontend on how to dynamically construct and render the personalized virtual exhibition scene.

### 2.4. Agent-Environment Communication: The SceneAction Protocol

The final output of the agent’s core logic is a standardized, declarative command called a SceneAction. This JSON object serves as a decoupled protocol between the backend intelligence and the frontend rendering engine. To more concretely illustrate the structure and function of this protocol, we have formalized it as pseudocode (see Algorithm 3).
**Algorithm 3** Structure of the SceneAction Communication Protocol  1:             ▹ This protocol defines the data structure for agent-to-frontend communication.   2:  **Structure: SceneAction**                                                  ▹ The root object of the command.  3:      action_type: String            ▹ A declarative command, e.g., “SHOW_INFO_GROUP”.  4:      payload: Payload                                 ▹ Contains the rich content curated by the agent.   5:  **Structure: Payload**;                                            ▹ The data core, output by the IDC Funnel.  6:      narrative: String (Optional)       ▹ The LLM-generated story to be displayed/spoken.  7:      layout_guidance: String (Optional)      ▹ LLM’s suggestion for spatial arrangement.  8:      items: List of SceneItem (Optional)               ▹ A list of multi-modal items to display.  9:      metadata: Dictionary (Optional)                 ▹ Any other relevant data for the frontend. 10:  **Structure: SceneItem**                 ▹ Represents a single multi-modal entity in the scene.11:      id: String                                      ▹ Unique identifier for the item (e.g., species ID).12:      name: String                                                       ▹ Display name (e.g., “Crested Ibis”).13:      image_url: String                                                   ▹ URL for the primary image asset.14:      audio_url: String                                                ▹ URL for the associated audio clip.15:      details: Dictionary                                  ▹ Other structured data (e.g., habitat, diet).

The core of this protocol is the SceneAction structure, which consists of two key fields: action_type and payload. The action_type is a declarative command (e.g., “SHOW_INFO_GROUP”) that instructs the frontend on the type of scene or interaction to render, providing a clear and finite set of capabilities for the frontend to implement.

The true richness of the experience is encapsulated within the payload. This data structure is the direct output of the IDC Funnel’s creative synthesis stage. As defined in the pseudocode, the payload can contain a narrative (the LLM-generated story to connect the items), layout_guidance (the LLM’s suggestion for spatial arrangement), and a list of SceneItem objects. Each SceneItem contains the multi-modal assets—such as images, audio clips, and detailed metadata—for a single entity (e.g., a bird species).

For example, after the agent processes a user’s request to “display Asian endangered birds,” it constructs a SceneAction. The action_type would be “SHOW_INFO_GROUP”. The payload would contain a generated narrative about these birds, layout guidance suggesting a specific arrangement, and a list of SceneItem objects, each corresponding to a bird that meets the criteria. This structured, yet flexible, protocol ensures reliable communication while enabling the on-the-fly creation of rich, dynamic, and multi-modal virtual scenes, effectively translating the agent’s curated plan into a tangible user experience.

## 3. Experiments

To rigorously evaluate our proposed SGAA and IDC Funnel, we designed and conducted a comprehensive, multi-faceted study. Our primary objective was to provide empirical evidence for a fundamental paradigm shift: from static information retrieval to dynamic, personalized experience curation in virtual environments. The evaluation combines (1) Component-Level Technical Benchmarks to validate our architecture’s performance against other agent paradigms, and (2) a Holistic User Study to measure the real-world impact on user experience and engagement.

### 3.1. Experimental Setup

**Knowledge Base:** The foundation of our knowledge base is authentic UAV sensor data collected from the Lalu Wetlands. To enrich this core dataset and robustly stress-test the system’s scalability, we augmented our collection using the public ‘BIRDS-525 Species’ dataset [26]. This process was twofold: (1) Asset Enhancement, where we matched standardized multi-modal assets from the public dataset to the species already identified in our UAV data, and (2) Categorical Expansion, where we incorporated the additional species from the dataset to achieve a larger scale. This resulted in a comprehensive knowledge base of 525 species that combines the relevance of real-world UAV data with the diversity of a large-scale public dataset. For each species, the knowledge base contained multi-modal data, including scientific and common names, images, and where available, audio recordings of calls, and structured textual information covering habitat, diet, conservation status, and unique behaviors.

**Figure 4:**
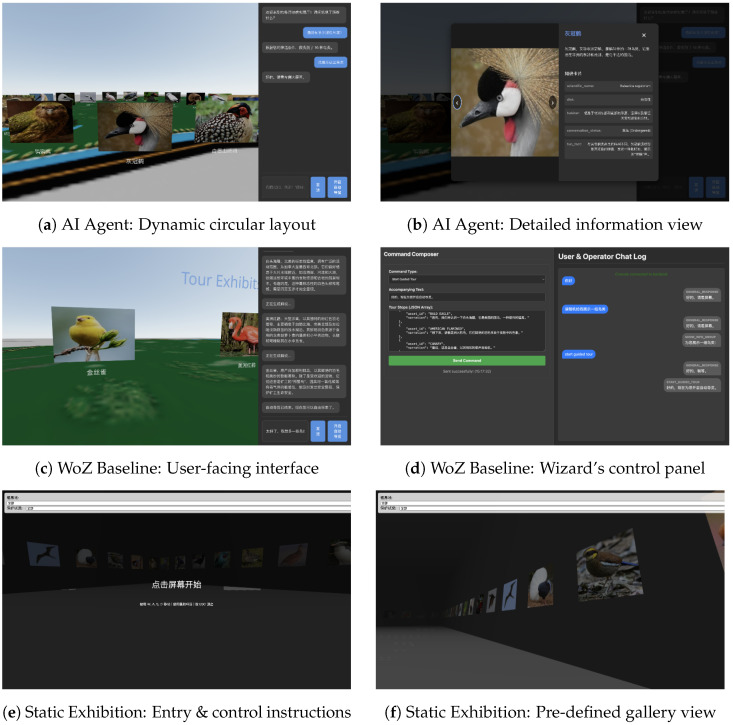
The user interfaces and system components for the three experimental conditions. Top Row (CurationAgent): (**a**) The system dynamically generates a personalized circular layout in response to user intent. (**b**) Users can select an item to view its detailed information card. Middle Row (WoZ Baseline): (**c**) The participant interacts with an interface visually identical to the AI Agent. (**d**) A human expert (the “wizard”) uses a separate backend control panel to manually simulate the agent’s responses. Bottom Row (Static Exhibition): (**e**) The system presents users with fixed keyboard navigation instructions upon entry. (**f**) Participants explore a pre-defined, static gallery of all items.

**System Implementations for Comparison:** To rigorously evaluate our framework, we implemented and compared three distinct systems for the user study. The key interfaces and components of these systems are detailed in Figure 4.

**CurationAgent:** This is the fully implemented agent based on our SGAA and IDC frameworks. As shown in Figure 4a, the system can translate a user’s conversational intent into a dynamically generated, personalized virtual layout (e.g., a circular arrangement of birds). Upon selecting an individual item, a detailed information card is presented (Figure 4b). The core LLM used for the “slow path” was Qwen2.5-72B-Instruct [22], an open-source model developed by the QwenLM project.

**User Study Baseline 1:** Wizard-of-Oz (WoZ [27]): Serving as the “gold standard” for interaction quality, this baseline simulated a perfect AI using a human expert. The participant interacted with a user-facing interface that was visually identical to our AI Agent system (Figure 4c). This design isolates the effect of system intelligence and performance by keeping the visual design constant. In the background, a human expert (the “wizard”) monitored the user’s dialogue and used a dedicated backend control panel (Figure 4). This panel provided the expert with a series of pre-defined actions, templates, and input fields. The expert’s task was to interpret the user’s intent and select the appropriate action and parameters; the control panel would then automatically generate and send the corresponding JSON command. This setup allowed the expert to focus on high-level decision-making, minimizing manual input latency and ensuring the WoZ baseline represented a true gold standard for interaction quality and intelligence.

**User Study Baseline 2:** Static Exhibition: This system represents the traditional, non-interactive paradigm and served as our primary baseline. Upon starting, it first presents the user with a screen detailing the fixed keyboard controls for navigation (e.g., W/A/S/D) (Figure 4e). Afterwards, the user enters and explores a pre-defined virtual gallery where all informational items are arranged in a static, linear sequence (Figure 4f).

**Technical Baseline 1:** Monolithic LLM Agent: An end-to-end agent without FSM or caching, receiving the full chat history on each turn. Used for technical benchmarks on responsiveness and reliability.

**Technical Baseline 2:** Standard RAG Agent (Ablation Baseline): To rigorously isolate the architectural contribution of the IDC Funnel, this baseline functions as an ablation variant sharing the identical technology stack (LLM, Embeddings, Knowledge Base) as the CurationAgent (detailed in Section 2.2). The critical distinction is that it bypasses the IDC’s ‘Intent Formalization’ and ‘Structured Filtering’ stages, relying exclusively on standard vector similarity search.

**Participants:** 27 volunteers (15 male, 12 female; aged 22–41 years) from the research institution and partner organizations participated in the study. All had computer science backgrounds and joined voluntarily without compensation. While the ultimate target audience for virtual exhibitions includes the general public, this specific group was selected to ensure high digital literacy and familiarity with conversational interfaces. Given the complexity of LLM-based interactions, participants with technical backgrounds act as ‘expert users’ who can effectively stress-test the system’s architectural responsiveness and logical consistency, minimizing friction caused by basic operational unfamiliarity during this prototype validation phase.

### 3.2. Component-Level Technical Benchmarking

Before the user study, we performed controlled tests to quantitatively validate the performance of our core architectural components.

#### 3.2.1. Responsiveness & Cost-Effectiveness (Fast Path Validation)

**Objective & Task:** To quantify the latency and cost advantages of the SGAA’s “fast path”, we executed a fixed 20-turn script, comprising 15 simple commands and 5 complex queries, on both CurationAgent and the Monolithic LLM Agent.

**Results:** The performance evaluation revealed the dual advantages of the SGAA framework in both speed and efficiency. For the 15 simple commands, CurationAgent’s “fast path” demonstrated near-instantaneous responsiveness, with an average latency of just 0.1 ms. This is in stark contrast to the Monolithic LLM Agent, which required an average of 2761 ms to process the same commands. To assess overall performance in a realistic mixed-use scenario, we analyzed the average latency across the entire 20-turn script. Here, CurationAgent maintained a superior average latency of 1512 ms, significantly outperforming the baseline’s 4301 ms. This efficiency is further underscored by the number of API calls: CurationAgent invoked the LLM only 5 times, whereas the baseline required 20 calls, confirming the architecture’s substantial cost-effectiveness.

#### 3.2.2. Reliability & Task Success Rate (FSM Guardrail Validation)

**Objective & Task:** To rigorously evaluate the FSM’s ability to act as a procedural “guardrail”, we designed a stress test involving a long-horizon, sequential task prone to context loss. The task required the agent to: (1) respond to the command “Please display all endangered birds,” (2) start a tour of the resulting list, (3) allow for one or more interruptions with questions at various points during the tour, and (4) correctly resume and successfully complete the remainder of the tour until the final item. We executed this full, interrupted tour task 100 times on both CurationAgent and the Monolithic LLM Agent.

**Success Criteria:** A run was deemed successful only if the agent, after any and all interruptions, correctly resumed the tour from the proper next item and proceeded sequentially through all remaining items until the end. Any deviation from the correct sequence at any point after an interruption was classified as a failure.

**Results:** The results revealed a stark difference in reliability. CurationAgent achieved a 95% task success rate, demonstrating high procedural integrity over long interactions. In contrast, the Monolithic LLM Agent, which lacks an explicit state management mechanism, only achieved a 57% success rate. The baseline’s failures were overwhelmingly due to context loss. A typical failure case occurred when, in response to the “please continue” command after an interruption, the baseline agent would randomly select a bird to continue the tour, completely breaking the logical sequence. This directly demonstrates that the FSM’s explicit state management is crucial for maintaining reliability in long-horizon interactions, a task where the monolithic agent’s reliance on unstructured context proved highly unstable.

#### 3.2.3. Precision on Complex Queries (Hybrid IDC-RAG Validation)

**Objective & Task:** This experiment was designed to quantify the critical contribution of our Hybrid IDC-RAG Funnel. As established in our methodology (Section 2.3.2), we hypothesized that Standard RAG Agents fail on complex queries because their probabilistic, vector-search mechanism cannot make definitive logical decisions. We evaluated our CurationAgent (using the hybrid IDC-RAG funnel) against a Standard RAG Agent on our curated dataset of 55 complex logical queries to empirically validate this hypothesis.

**Results:** The results provided a stark and conclusive validation of our approach. Our CurationAgent (Hybrid IDC-RAG) achieved a Precision of 85.5%. This high level of precision is the direct result of our hybrid architecture: the IDC component’s initial “Intent Formalization” successfully translated the queries’ logical constraints, and the “Filtered Augmentation” stage ensured that even the semantically retrieved RAG results were re-filtered to adhere to these strict logical rules.

In dramatic contrast, the Standard RAG Agent achieved only 52.7% Precision. Qualitative Analysis of RAG Failure and IDC Correction: To contextualize these metrics, we analyzed specific failure cases where the hybrid architecture demonstrated superiority. A representative example involves the query: “Show me endangered birds.”

- **Standard RAG Failure (False Positive):** The baseline RAG agent incorrectly retrieved the Bar-headed Goose (Conservation Status: Least Concern). This occurred because its textual description contained the sentence *“This species is currently not considered endangered.”* The vector embedding mechanism captured the high semantic similarity with the keyword “endangered” but failed to parse the negation logic (“not”), resulting in the retrieval of a non-endangered species.
- **IDC Success (Logical Elimination):** In contrast, the CurationAgent’s IDC Funnel correctly extracted the structured filter {“conservation_status”: “Endangered”}. During the Conditional Re-filtering stage, the system checked the metadata of the RAG-retrieved Bar-headed Goose. Since its status (Least Concern) did not match the strict filter (Endangered), the IDC logic explicitly eliminated this false positive result. This demonstrates that structured intent extraction is essential for ensuring logical correctness where probabilistic embedding fails.

This result confirms that for high-precision, logic-based tasks, a standard RAG approach is insufficient, and our hybrid IDC-RAG funnel provides the necessary mechanism for robust logical filtering.

### 3.3. Holistic User Study

Building on the technical validation, this study assesses the overall impact of our system on real users.

#### 3.3.1. Experiment 1: Paradigm Validation

**Research Question:** Does a dynamic, conversational agent provide a measurably superior user experience compared to a static exhibition?

**Procedure & Metrics:** Participants were asked to complete a standardized task (“Find three endangered, forest-dwelling bird species and their primary diet”), while we measured Task Completion Time, Cognitive Load (NASA-TLX [28]), and System Usability (SUS [29]). To avoid ambiguity, this Task Completion Time metric specifically measures the system’s response latency: the time from the user submitting the final query to the system first displaying the correct results, not the user’s entire end-to-end task duration.

**Results:** The results for our paradigm validation are summarized in Table 1. The interactive paradigms were clearly superior to the static exhibition across all key metrics.

A one-way ANOVA confirmed a significant effect of the system paradigm on all three metrics. For Task Completion Time (F(2,24)=7247.95,p<0.001), post-hoc analysis revealed a clear hierarchy: the Static Exhibition was by far the slowest (169.04 s). More importantly, CurationAgent (2.91 s) was significantly faster than even the human-expert WoZ baseline (15.23 s), demonstrating exceptional efficiency.

Similarly, for Cognitive Load (NASA-TLX) (F(2,24)=318.42,p<0.001), the Static Exhibition (65.56) imposed the highest cognitive load. Notably, CurationAgent (17.78) imposed the lowest load, performing significantly better (i.e., lower) than the WoZ baseline (25.35).

Finally, for System Usability (SUS) (F(2,24)=19.02,p<0.001), CurationAgent (M=74.67,SD=5.57) and the WoZ system (M=68.89,SD=5.21) were both rated as significantly more usable than the Static Exhibition (M=59.33,SD=5.20).

#### 3.3.2. Case Study: Architectural Validation of Complex Intent and Robustness

To qualitatively validate the architectural advantages of the CurationAgent, which are not fully captured by the user study in Experiment 1, we conducted a case study analysis on three representative, challenging queries. This analysis demonstrates the agent’s core mechanisms for (1) complex intent parsing, (2) multi-step programmatic execution, and (3) robust guardrailing.

**Case 1: Complex Boolean Logic Query**

User Query 1:

*“I am looking for a bird found in (North America OR Europe), which must be a (small songbird), AND its conservation status is (NOT Endangered) AND (NOT Vulnerable).”*

**Analysis and Mechanism:** This query is challenging as it requires parsing nested Boolean logic (AND, OR, NOT), which typically fails standard keyword or simple vector-based RAG systems.

The CurationAgent’s Intent Parsing Module addresses this by leveraging an LLM for structured output generation. The natural language query is not merely embedded; it is parsed into a standardized JSON object that explicitly defines the multi-layered filtering logic (see Listing 2). This structured query is then passed to the Data Processing Module, whose internal query engine can deterministically execute the complex ‘AND/OR/NOT_EQUALS’ logic against the indexed knowledge base.

**Listing 2.** Structured JSON Query Parsed by the LLM.

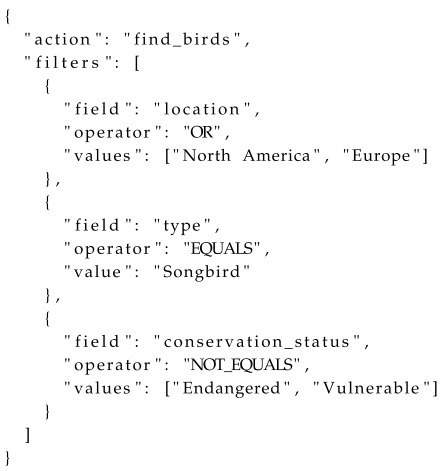



**Case 2: Multi-Step Procedural Workflow**

User Query 2:

*“First, find all birds that live in the forest. THEN, exclude all (finches and crows) from that list. FINALLY, from the remaining results, show me only those that are (endangered) OR (have colorful, i.e., non-monochrome, feathers).”*

**Analysis and Mechanism:** This query is not a single atomic request but a programmatic workflow that demands planning, state management, and sequential execution. Atomic RAG systems are incapable of handling such instructions.

The CurationAgent’s SGAA manages this complexity. It uses the LLM as a planner (via Chain-of-Thought or Tool Calling) to decompose the instruction into an Execution Plan:

- **Step 1 (Retrieve):** Get_birds(Habitat = “Forest”) -> Result_Set_1;
- **Step 2 (Filter/Exclude):** Filter_birds(input = Result_Set_1, exclude_families = [“Fringillidae”, “Corvidae”]) -> Result_Set_2;
- **Step 3 (Final Filter):** Filter_birds(input = Result_Set_2, conditions = [“status”: “Endangered”, “color”: “colorful”]) -> Final_Result.

The engine maintains the session state, passing the intermediate results (Result_Set_1, Result_Set_2) between steps, effectively executing the user’s complex intent as a procedure.

**Case 3: Robustness and Guardrails**

User Query 3:

*“Tell me a joke,” or “What is the weather in Beijing?”*

**Analysis and Mechanism:** A robust agent must maintain its defined role and not waste resources on out-of-scope (OOS) requests. The CurationAgent employs an LLM-based Intent Classifier as a preliminary check.

This classifier, primed with the agent’s specific role (a “Lalu Wetland virtual exhibition guide”), identifies the user’s intent as “Out-of-Scope” with high confidence. Upon this detection, the SGAA bypasses the entire data processing workflow and triggers a graceful rejection policy, responding:

*“I’m sorry, I am an assistant focused on the Lalu Wetland virtual exhibition and do not provide jokes or weather information. Would you like to know about the local flora or fauna?”*

This guardrail mechanism ensures system robustness, focuses the interaction, and maintains the agent’s professional persona.

### 3.4. Summary of Experimental Findings

Our comprehensive evaluation, combining rigorous technical benchmarks with a multi-faceted user study, provides compelling evidence for the superiority of our proposed framework. The technical benchmarks quantitatively confirmed that our core components are significantly more responsive, reliable, and precise than standard agent architectures. Building upon this, the paradigm validation (Section 3.3.1) demonstrated that these component-level advantages translate directly into a measurably superior user experience (e.g., lower task time, reduced cognitive load, and higher usability) compared to traditional paradigms, while our case study (Section 3.3.2) further validated the architecture’s robustness in handling complex, multi-step queries.

## 4. Discussion

This study addresses the fundamental contradiction between the static nature of virtual exhibitions and the dynamic, personalized needs of modern users. Our findings demonstrate that the CurationAgent, by integrating the State-Gated Agent Architecture (SGAA) with the Intent-Driven Curation (IDC) Funnel, successfully resolves this conflict. The system achieved a 95% task success rate and an average latency of 1.5 s (with sub-millisecond response for high-frequency interactions), significantly outperforming general-purpose baselines. Below, we discuss these findings through cognitive theory, comparative analysis, and practical feasibility.

### 4.1. Theoretical Implications: Validating Dual Process Theory

Our empirical results offer strong validation for applying Dual Process Theory [19] to agent design. The performance disparity (95% success for CurationAgent vs. 57% for the baseline) suggests that monolithic LLMs suffer from a “cognitive bottleneck” when simultaneously handling fast, instinctive interactions (System 1) and slow, complex reasoning (System 2). By enforcing a structural “division of labor”—where the deterministic FSM acts as a System 1 “guardrail” and the LLM functions as a focused System 2 reasoning engine—SGAA effectively operationalizes cognitive efficiency. This confirms that for robust task-oriented HCI, hybrid architectures combining deterministic control with stochastic generation are a necessity, not merely an optimization.

### 4.2. Comparative Analysis: Logic over Probability

Beyond efficiency, our work challenges the reliance on standard Retrieval-Augmented Generation (RAG) for complex tasks. While RAG [25] excels at semantic proximity, our benchmarks revealed its limitation in handling logical constraints (52.7% precision vs. 85.5% for IDC). The standard RAG agent’s failure to distinguish “endangered” from “not endangered” due to vector similarity highlights the risks of purely probabilistic retrieval. In contrast, the IDC Funnel’s “Intent Formalization” bridges natural language ambiguity and database rigidity. This signals a paradigm shift from “Information Retrieval” (finding likely answers) to “Experience Curation” (constructing logically valid narratives), prioritizing structured intent understanding over simple vector alignment.

### 4.3. Practical Deployment: Latency and Resource Trade-Offs

To evaluate real-world feasibility, we analyzed the trade-offs between two deployment paradigms. Cloud-based deployment (utilized in our prototype via Qwen2.5-72B API) offers a flexible pay-as-you-go model (∼¥4.00 RMB/1 M tokens), significantly enhancing cost-efficiency by eliminating the need for expensive on-site high-performance computing hardware. While raw cloud inference naturally incurs network and processing overhead, our SGAA architecture effectively mitigates these latencies through “Fast Path” caching and FSM guardrails, achieving a responsive average latency of 1.5 s (as validated in Section 3.2.1). Conversely, local deployment (e.g., theoretically utilizing Int4 quantized models on dual RTX 3090s) prioritizes data privacy and stability but incurs higher initial hardware costs. Consequently, the framework supports adaptable deployment strategies: the cloud approach provides a scalable solution for venues with reliable connectivity, while local inference remains a viable, high-performance alternative for privacy-sensitive or connectivity-constrained venues, such as remote wetland field stations.

### 4.4. Limitations and Future Directions

Despite promising results, several limitations delineate future research avenues. First, our framework currently relies on a pre-structured knowledge base; future work should integrate unstructured multi-modal ingestion to automate knowledge updates. Second, the FSM is hand-authored, limiting scalability; exploring semi-automatic state learning from interaction logs could enhance adaptability. Third, our single-session study necessitates longitudinal research to investigate long-term user adaptation. Finally, future iterations should expand beyond text to include multi-modal inputs (e.g., gaze, gesture) within the SGAA’s Fast Path, enabling more natural and intuitive navigation.

## 5. Conclusions

This paper introduced and empirically validated CurationAgent, a novel agent-driven framework for the on-the-fly construction of personalized virtual spaces, demonstrating a superior alternative to both static virtual exhibitions and general-purpose LLM agents. Through the design of CurationAgent’s core components—the SGAA and the IDC Funnel—we have demonstrated a viable and superior path forward. Our comprehensive experiments show that by intelligently orchestrating the capabilities of LLMs within CurationAgent’s robust, state-aware architecture, it is possible to create interactive experiences that are not only fast, reliable, and precise, but also demonstrably more effective at engaging users. This work represents a significant step towards a future where virtual environments are no longer static repositories of information, but dynamic, intelligent systems for exploration and discovery.

## Figures and Tables

**Table 1:** Results for Paradigm Validation (Experiment 1). Values are reported as Mean (SD).

Metric	CurationAgent	Wizard-of-Oz (WoZ)	Static Exhibition
Task Completion (s)	2.91 (0.95)	15.23 (2.31)	169.04 (72.72)
Cognitive Load (TLX)	17.78 (3.08)	25.35 (6.94)	65.56 (5.96)
System Usability (SUS)	74.67 (5.57)	68.89 (5.21)	59.33 (5.20)

## Data Availability

The original contributions presented in this study are included in the article. Further inquiries can be directed to the corresponding author.
